# Supplementary material for: Prospective Evaluation of Neoadjuvant Imatinib Use in Locally Advanced Gastrointestinal Stromal Tumors: Emphasis on the Optimal Duration of Neoadjuvant Imatinib Use, Safety, and Oncological Outcome
Source: Cancers (Basel). 2019 Mar 25;11(3):424. doi: 10.3390/cancers11030424 (PMC6468640; doi:10.3390/cancers11030424)
Supplement: Supplementary file 1 [file cancers-11-00424-s001.pdf]

Supplementary Materials: Prospective Evaluation of Neoadjuvant Imatinib Use in Locally Advanced Gastrointestinal Stromal Tumors: Emphasis on the Optimal Duration of Neoadjuvant Imatinib Use, Safety, and Oncological Outcome

Shang-Yu Wang, Chiao-En Wu, Chun-Chi Lai, Jen-Shi Chen, Chun-Yi Tsai, Chi-Tung Cheng, Ta-Sen Yeh and Chun-Nan Yeh

Table S1. Mutation Analysis.

Mutation Patterns	Whole Cohort (*n* = 51)	Per protocol Patients (*n* = 40)	Per protocol Patients with OP (*n* = 27)
KIT mutation			
Exon 11	25	17	16
Exon 9	1	1	1
Exon 13	1	1	1
Exon 11 + Intron 10	3	2	2
Exon 11 + 9	1	1	1
Exon 9 + Intron 10	1	1	1
PDGFRA			
Exon 18 (D842V)	1	0	0
Wild type	4	3	2
ND	14	14	3

PDGFRA: platelet-derived growth factor receptor A. ND: mutation analysis not performed.

© 2019 by the authors. Licensee MDPI, Basel, Switzerland. This article is an open access article distributed under the terms and conditions of the Creative Commons Attribution (CC BY) license (http://creativecommons.org/licenses/by/4.0/).
